# Immune system dynamics in response to *Pseudomonas aeruginosa* biofilms

**DOI:** 10.1038/s41522-025-00738-2

**Published:** 2025-06-12

**Authors:** Abhijeet Sahu, Rohit Ruhal

**Affiliations:** School of Bio Science and Technology, VIT Vellore, Vellore, Tamil Nadu India

**Keywords:** Biofilms, Microbial communities

## Abstract

*Pseudomonas aeruginosa* biofilms contribute to chronic infections by resisting immune attacks and antibiotics. This review explores how innate immunity, including neutrophils, macrophages, and dendritic cells, responds to biofilms and how adaptive mechanisms involving T cells, B cells, and immunoglobulins contribute to infection persistence. Additionally, it highlights immune evasion strategies and discusses emerging therapies such as immunotherapy, monoclonal antibodies, and vaccines, offering insights into enhancing biofilm clearance and improving treatment outcomes.

## Introduction

The human immune system is a sophisticated defence system which can identify and neutralize pathogenic invaders while continuing to be tolerant to self-antigens. However, certain bacterial pathogens have evolved intricate strategies to bypass immune detection and destruction, making infections particularly challenging to treat. Among these organisms*, Pseudomonas aeruginosa* stands out as a particularly virulent and versatile microbe. It is accountable for a broad range of infections, notably in persons with impaired immune systems, such as those having persistent chronic wounds, cystic fibrosis (CF), or immunosuppression due to diseases or medical interventions^1^. The virulence of *P. aeruginosa* is multifaceted, involving a variety of virulence components, but perhaps the most insidious of its strategies is its capability to form biofilms. Organized groups of bacteria called biofilms are encased in extracellular polymeric substance (EPS) that the bacteria produce on their own, giving them a protective habitat^2^. These biofilms are not just random bacterial aggregates but highly organized systems that provide a survival advantage to the bacteria within them. The matrix of biofilm operates as an environmental barrier, safeguarding bacterial cells from outside stresses like the host’s immunological system responses and antimicrobial agents^3^. This biofilm growth mode not only enhances *P. aeruginosa* survival in adverse conditions, but also contributes to its persistence in the host by conferring resistance to both antimicrobial agents and immune system attacks^4^. Infections caused by biofilms are notoriously challenging to cure due to the inadequate absorption of antibiotics through the EPS matrix, the existence of persister cells, which are naturally resistant to antibiotics, and the way bacteria in biofilms adapt their metabolism^5^.

Most research on the immune response to bacterial infections has traditionally centred on planktonic bacteria. Consequently, we understand very little regarding how the body’s immune system responds to infections caused by biofilms. On the other hand, new in vitro and in vivo research has begun to clarify how the innate and adaptive immunological systems respond to bacteria within biofilms^6^. The manner in which the immune system reacts to *P. aeruginosa* biofilms is complex and constantly evolving, relying on a combination of both innate and adaptive immunological reactions. When exposed to a biofilm, both neutrophils and macrophages are drawn to the point of infection. There, they engage in phagocytosis, generate ROS, and produce antimicrobial peptides in an effort to eradicate the bacteria^7^. However, the biofilm’s EPS matrix can hinder the effectiveness of these immune responses, resulting in inadequate bacterial clearance and the development of a persistent infection^8^. Histidine kinase LadS, paired with the response regulator PA0034, enhances expression of the essential chaperone-usher pathway (CUP) pilus, cupA, in *P. aeruginosa*. However, the LadS/PA0034 system is disrupted by reactive oxygen species (ROS) from macrophages (MΦs). Upon detecting ROS, the bacteria downregulate cupA pilus expression, shifting to a less adhesive state that reduces MΦ phagocytic efficiency by decreasing bacterial adhesion. Essentially, when MΦ-derived ROS are sensed, the LadS/PA0034 pathway is inhibited, and the bacteria adopt a poorly adhesive phenotype, helping them evade phagocytic clearance in acute infections^9^. Biofilm-derived Membrane vesicles (b-MVs) from *P. aeruginosa* PAO1 elicit a stronger immune response in macrophages than planktonic-derived MVs, particularly by increasing proinflammatory cytokines like Il1b, Il6, and Il12p40^10^.

Moreover, *P. aeruginosa* within the biofilm can alter its phenotype, becoming less susceptible to immune-mediated killing and more adept at evading immune surveillance^11^. Furthermore, *P. aeruginosa* biofilms can alter the immunological system of an individual in ways that benefit their own survival. The biofilm’s protective matrix serves as a physical shield, making it harder for immune cells to access the bacteria. On top of that, certain elements of the EPS matrix, like alginate, can interfere with the immune response by blocking phagocytosis and decreasing the release of cytokines that promote inflammation^12^. This immune evasion is further compounded by the bacteria’s ability to secrete virulence factors that degrade immune components and disrupt signalling pathways essential for effective immune responses^13^. These interactions not only enable *P. aeruginosa* to survive inside the host, but they also serve an integral part in the pathophysiology of persistent infections, which are marked by tissue damage, prolonged inflammation, and poor recovery^14^. The long-term prevalence of *P. aeruginosa* biofilms in chronic infections is a significant hurdle to both the body’s immune system and accessible medical treatments. Chronic inflammation triggered by persistent biofilms can result in increasing tissue damage and organ failure, as seen in the lungs of individuals with CF, where a persistent *P. aeruginosa* infection is a leading cause of morbidity and death^15^. The immune system’s inability to efficiently remove biofilms results in the creation of new therapeutic approaches, which either boost the immune system or specifically target the structures of biofilms^16^.

The goal of the review article is to present an extensive overview of the present level of knowledge about the dynamics of the immune system in reaction to *P. aeruginosa* biofilms. By exploring the immune evasion and the resultant immune responses, this paper will address possible therapeutic intervention strategies and draw attention to the difficulties presented by biofilm-based infections. The review will draw upon the latest research to offer insights into how the immune system interacts with biofilms, and how these interactions can be leveraged or modulated to improve clinical findings for patients suffering from persistent *P. aeruginosa* infections.

## Innate immune mechanisms in response to *P. aeruginosa* biofilm infections

The body’s primary form of defence towards diseases is the innate immunological system, which is triggered as soon as a pathogen is encountered. This defence mechanism is non-specific and relies on germline-encoded, non-clonal cellular and humoral responses, which function without prior exposure to the pathogen^17^. Major elements of the inherent immune reaction in *P. aeruginosa* biofilm-associated infections are the neutrophils, dendritic cells, macrophages, complement system, and natural killer (NK) cells. Experiments have demonstrated that an intense immune reaction is elicited when human neutrophils and macrophages are exposed to *P. aeruginosa* biofilms that are devoid of planktonic microorganisms^18^. This comprises phagocytosis, cytokine generation, respiratory burst, biofilm penetration, neutrophil buildup, and finally, the elimination of biofilm bacteria. Higher levels of bacterial aggregation in *P. aeruginosa* cultures cause neutrophils to undergo a more severe respiratory burst and macrophages to release more cytokines^19^. In vivo research shows a notable buildup of active neutrophils in the lungs, as demonstrated by initial sampling of mice lungs affected with biofilms of *P. aeruginosa*^20^. Similar neutrophil responses are seen in experimental models that have persistent wounds infected with *P. aeruginosa* biofilms^21^. The relevance of each innate immune component in *P. aeruginosa* biofilm-associated infections is discussed in Table 1.

**Table 1:** Immune components and their role in *Pseudomonas aeruginosa* biofilm infections

Immune component	Role in immune response	Interaction with biofilm	Mechanism of action	Impact on biofilm persistence	References
Neutrophils	Act as the first responders by engulfing pathogens and releasing antimicrobial peptides.	Neutrophils can become trapped at the biofilm periphery, where the biofilm matrix hampers their access to the bacteria.	Phagocytosis of free bacteria and production of reactive oxygen species (ROS) to combat infection.	Inability to penetrate biofilms allows bacteria to survive, leading to persistent inflammation and tissue damage.	202 203 204
Macrophages	Responsible for engulfing pathogens and initiating inflammation through cytokine production.	Biofilm matrix blocks efficient macrophage activity, limiting their ability to digest biofilm-embedded bacteria.	Engulf and destroy bacteria while signaling other immune cells to enhance the immune response.	Ineffective biofilm clearance leads to prolonged inflammation and promotes chronic infection.	19 205
Dendritic cells (DCs)	Link innate and adaptive immunity by presenting antigens to T-cells, initiating adaptive immune responses.	Biofilm components, including quorum-sensing molecules, can inhibit DC activation, limiting antigen presentation to T-cells.	Capture and present antigens to T-cells, activating adaptive responses essential for long-term infection control.	Impaired antigen presentation prevents effective T-cell activation, contributing to biofilm persistence.	6 112 206
T cells (Th1, Th2, Th17)	Essential for adaptive immunity, with Th1 cells aiding pathogen clearance and Th2/Th17 cells promoting inflammation.	In biofilm infections, Th2/Th17 responses dominate, leading to an inflammatory environment rather than effective pathogen clearance.	Release cytokines (e.g., IL-17, IFN-γ) that recruit other immune cells and promote antibody production.	Imbalanced cytokine responses contribute to excessive inflammation, worsening tissue damage without resolving the infection.	207 208 209
B cells & antibodies (IgA, IgG)	Produce antibodies that help in bacterial clearance by targeting bacterial antigens and facilitating phagocytosis.	Antibodies struggle to penetrate the biofilm matrix, reducing opsonization and complement activation.	Opsonization of bacteria for phagocytosis and neutralization of toxins at mucosal surfaces.	Incomplete biofilm clearance leads to persistent infection and immune complex formation, exacerbating inflammation.	6 101 138
Cytokines/chemokines	Signal and recruit immune cells to infection sites, including IL-8 (neutrophil recruitment) and IL-17 (inflammation).	Biofilm-induced dysregulation of cytokine production perpetuates immune cell recruitment without resolution.	Recruit neutrophils, macrophages, and lymphocytes to control infection and maintain inflammation to eliminate pathogens.	Chronic cytokine production promotes prolonged inflammation and tissue damage, allowing biofilm persistence.	210 211
Pattern recognition receptors (PRRs)	Detect pathogen-associated molecular patterns (PAMPs), such as LPS, flagellin, and bacterial DNA, to initiate immune responses.	Biofilm matrix components, like alginate, may mask PRR ligands, reducing their ability to detect biofilm bacteria.	Initiate immune responses by recognizing PAMPs and activating downstream pathways, including cytokine release.	Decreased PRR activity leads to immune evasion, enabling the biofilm to persist and cause chronic infection.	6 71 212
Complement system	Facilitates pathogen lysis and enhances phagocytosis by immune cells.	Biofilm matrix components interfere with complement activation, shielding bacteria from opsonization and direct lysis.	Triggers opsonization and formation of membrane attack complexes to lyse bacteria.	Limited complement activity in biofilms enables bacterial survival, contributing to long-lasting infections.	121 213 214
Natural killer (NK) cells	Target and kill infected cells and bacteria through cytotoxic activity and production of immune-modulating cytokines.	NK cell recognition of biofilm-embedded bacteria is impaired due to the biofilm’s protective barrier.	Release perforin and granzyme to kill infected cells and secrete cytokines that promote immune responses.	Reduced NK cell function supports biofilm survival and hinders effective infection resolution.	6 215 216
GM-CSF	Promotes the production of granulocytes and macrophages, enhancing innate immune responses.	Biofilms sustain high levels of GM-CSF, promoting excessive neutrophil recruitment, but biofilm barriers limit effective pathogen clearance.	Stimulates the proliferation and activation of neutrophils and macrophages.	Prolonged immune cell recruitment worsens inflammation without resolving biofilm infections.	6 217 218
Toll-like receptors (TLRs)	Recognize bacterial components like LPS, flagellin, and DNA, initiating inflammatory responses.	TLR signaling is inhibited by biofilm matrix components like alginate, reducing the immune system’s ability to recognize and respond to infection.	Activate immune responses by triggering cytokine production and recruiting immune cells to infection sites.	Impaired TLR signaling reduces immune detection and contributes to immune evasion by biofilm-embedded bacteria.	6 219
Virulence factors (e.g., pyocyanin)	Bacterial virulence factors, such as pyocyanin, can disrupt immune cell function and promote biofilm survival by generating oxidative stress and immune suppression.	Virulence factors secreted by biofilm-encased bacteria impair immune responses, including neutrophil function and cytokine signaling.	Inhibit immune cell activity, disrupt host signaling, and increase oxidative stress, promoting biofilm survival.	Virulence factors allow biofilm-embedded bacteria to evade immune clearance and establish chronic infections.	70 220

This table provides a concise overview of how key immune components interact with *Pseudomonas aeruginosa* biofilms, affecting their function and contributing to biofilm persistence. Neutrophils, trapped at the biofilm’s edge, are unable to clear bacteria effectively. Macrophages struggle with biofilm-embedded bacteria, causing chronic inflammation. Dendritic cells (DCs) are inhibited from presenting antigens, reducing T-cell activation. In biofilm infections, Th2/Th17 responses prevail, driving inflammation without clearing pathogens. B cells and antibodies have limited penetration, leading to incomplete bacterial clearance. Cytokines and chemokines are dysregulated, fueling prolonged inflammation. Pattern recognition receptors (PRRs) are masked, reducing bacterial detection. The complement system is blocked, limiting bacterial lysis. Natural killer (NK) cells and GM-CSF promote immune cell recruitment but fail to clear biofilms. Toll-like receptors (TLRs) are inhibited by biofilm barriers, and virulence factors suppress immune function, aiding biofilm survival.

## Innate immune response in CF patients with persistent *P. aeruginosa* lung infection

### Role of neutrophils in innate immune response

**Fig. 1:**
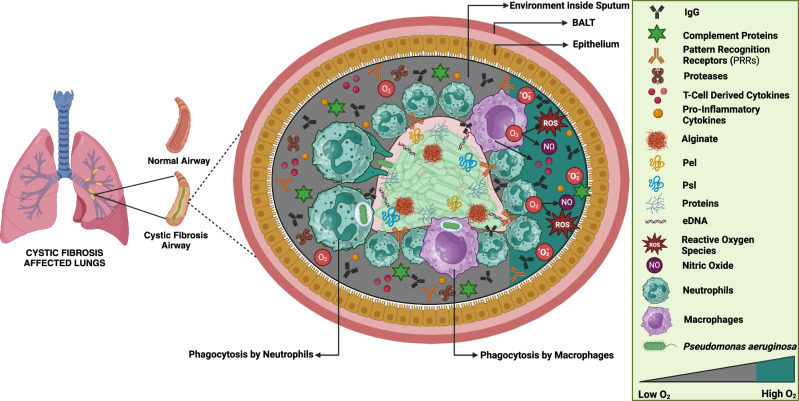
Innate immune response to *Pseudomonas aeruginosa* biofilms in cystic fibrosis lungs. This figure highlights the contrast between a healthy airway and the altered airway of a cystic fibrosis (CF) patient, where thick mucus facilitates the development of biofilms. The biofilm, primarily composed of *Pseudomonas aeruginosa*, is encapsulated by extracellular DNA (eDNA), proteins, and polysaccharides (alginate, *Pel*, *Psl*), which protect it from immune defences. Surrounding the biofilm, neutrophils and macrophages attempt to clear the infection through phagocytosis, though their actions are hindered by the biofilm’s structure. Additionally, reactive oxygen species (ROS) and nitric oxide (NO), key antimicrobial molecules, are depicted attacking the biofilm. Other important immune components include complement proteins, immunoglobulins (IgG), and pattern recognition receptors (PRRs), which recognize and attempt to neutralize the pathogen. The biofilm’s oxygen gradient, with lower oxygen levels at the center, further complicates the immune response. This image illustrates the complexity of biofilm persistence in CF airways, contributing to chronic lung infections.

CF patients with persistent *P. aeruginosa* lung infections rely significantly on their innate immune systems. The elevated buildup of neutrophils in the respiratory tract is intimately linked to declining lung function in these patients^22^. These neutrophils remain continuously active, producing respiratory bursts^23^ and nitric oxide^24^, leading to oxidative and proteolytic damage that significantly contributes to the gradual destruction of lung tissue in CF patients^25^. Alterations in the CFTR gene, which lead to faulty ion transport, exacerbate chronic *P. aeruginosa* infections of the lungs in individuals with CF^26^. *P. aeruginosa* biofilms predominate in CF patients’ infected lungs, where neutrophils gather around the biofilm and phagocytosis primarily engulfs a small number of free-floating bacteria^27^. This intense neutrophil response, along with the depletion of molecular oxygen due to the active respiratory burst, creates a hypoxic environment in the infected lungs^28^. In this low-oxygen setting, neutrophils rely heavily on anaerobic glycolysis for energy, consuming large amounts of glucose^29^ and contributing to the high L-lactate^30^ levels found in CF sputum. The innate immune response in CF patients is further influenced by various factors, including the interaction of neutrophils with bacterial components like lipopolysaccharides (LPS), antigen-antibody complexes and alginate^6^. The magnitude of this reaction may be heightened by priming with LPS and other innate immunological mediators such as leukotriene B4, TNF-α, IL-8, and platelet-stimulating factor^31^. As neutrophils migrate through inflamed tissue, their activation is further stimulated by interactions with integrins and inflammatory cytokines^32^. Neutrophils in CF patients appear to be functioning normally, which implies that non-CF patients may also have similar infections and responses to *P. aeruginosa* biofilms. A large buildup of neutrophils is commonly seen in infected prosthetic joints, and this response becomes even more intense in chronic wound and peritoneal infection models involving *P. aeruginosa* biofilms^20,33^. The biofilm lifestyle that *P. aeruginosa* and neutrophils produce during their in vitro interactions is probably crucial for the production of biofilms in organisms^34^. Neutrophils are essential for immunological defence, but they may not work as well in CF patients, in part because *P. aeruginosa* biofilms produce rhamnolipids. These rhamnolipids, regulated by quorum sensing (QS), can induce necrosis in neutrophils, thereby protecting the biofilm from immune attack^11^. *P. aeruginosa* capacity to draw neutrophils to the site of infection and then destroy them using rhamnolipids emphasizes the intricacy of the immune reaction during biofilm infections^35^. However, in CF patients, prolonged lung infection often leads to genetic adaptations in *P. aeruginosa*, including mutations in the QS regulator gene lasR^36^. These mutations can disrupt QS, leading to ineffective neutralization of chemotactic cytokines, which in turn attract more neutrophils and intensify lung inflammation^37^. Although aggregates larger than 5 μm can avoid neutrophil phagocytosis, the size of bacterial aggregates in biofilms also affects how well bacteria are protected from the immune system’s response^38^. A study found that *P. aeruginosa* RNA (RNAPAE) reduces immune responses in lung cells and neutrophils, which are key for bacterial defence. RNAPAE, especially short RNA fragments, decreased neutrophil activation and phagocytosis, aiding bacterial survival. These results highlight RNAPAE’s role in modulating immune responses in lung infections^39^. Figure 1 describes how the innate immune system reacts to *P. aeruginosa* biofilms in the lungs of people with CF.

### Role of PRRs in innate immune response

Pattern recognition receptors (PRRs) are utilised by the innate immune system to identify invasive microorganisms. These receptors attach to conserved microbial patterns called PAMPs, which set off the host’s immune system reaction. PRRs are typically found on cell surfaces, within cell membranes, in the cytoplasm, or as soluble receptors. One of the most well-known families of PRRs is the toll-like receptors (TLRs), some of which are present on the cell periphery and mainly detect microbial components such as proteins, lipids, and lipoproteins, while others are found inside cells and identify bacterial nucleic acids^40^. In the pulmonary system of CF patients with persistent infections, neutrophils express the Myd88-dependent receptor TLR5, which recognises flagellin, more often^41^. This is intriguing because *P. aeruginosa*, especially in its mucoid biofilm form found in CF lungs, typically lacks flagella. The upregulation of TLR5 may be influenced by factors like G-CSF, IL-8, TNF-α, and interactions between bacterial lipoproteins with TLR2 and TLR1^40^. While the contribution of TLR5 in infections caused by biofilm remains unclear, it might help neutrophils target planktonic *P. aeruginosa* that still possess flagella^42^. Moreover, free-floating *P. aeruginosa* tends to trigger a more powerful immune reaction than its biofilm structure, as seen by the enhanced IL-8 production by the epithelial cells^43^. Interestingly, bacterial eDNA, a key component of biofilms, may also activate neutrophils, enhancing IL-8 production without necessarily involving TLR9^44^. Bacterial extracellular DNA (eDNA) is crucial to biofilm structure, development, and resistance to host defences, with DNase I and DNase1L3 playing key immune roles in countering biofilm formation. eDNA’s Z-form, which resists DNase I, may allow bacteria to evade certain immune mechanisms, while DNA sensors like cGAS/STING and PRRs such as TLRs recognise eDNA and its complexes as biofilm-associated PAMPs. Targeting eDNA offers a possible therapeutic strategy for treating persistent infections linked to biofilms in light of these findings^45^.

### Role of alginate, *Pel* and *Psl* in innate immune response

One of the main virulence factors for persistent lung infections in individuals with CF is alginate, which is a crucial part of the biofilm matrix that mucoid *P. aeruginosa* produces^46^. It can enhance the respiratory burst of neutrophils and trigger cytokine production in monocytes, likely through activation via TLR2 and TLR4^47^. However, the receptors involved in neutrophil activation by alginate are still unclear. Other polysaccharides, such as *Psl* and *Pel*, are also present in the biofilm matrixome and support the innate immune response. *Psl* and alginate-rich biofilms are very good in inducing neutrophil activation^48^. It remains uncertain whether the immune response to these exopolysaccharides is stronger in biofilms compared to planktonic bacteria. *P. aeruginosa* exopolysaccharides, particularly Pel, perform an integral part in defending against oxidative stress from disinfectants like NaOCl and H_2_O_2_. Inactivation of both *Psl* and *Pel* significantly increased the bacterium’s vulnerability to these oxidants, underscoring *Pel*’s vital protective role. Additionally, co-culture biofilms with *P. aeruginosa* WT strains offered enhanced oxidative resistance, highlighting the EPS matrix’s function in safeguarding against environmental stressors^49^.

### Role of complement systems in innate immune response

The complement system’s role in biofilm infections is still not fully understood, as *P. aeruginosa* has evolved ways to avoid complement activation, such as producing elastase and alkaline protease, which inactivate complement components^50^. Alginate, particularly when O-acetylated, helps protect mucoid *P. aeruginosa* biofilms by preventing complement opsonization^51,52^. The *Psl* also plays a protective role by preventing mucus-like bacteria from being tagged and eliminated by complement proteins in human serum. Nevertheless, the activation of the complement system appears to be independent of biofilm formation, since planktonic bacteria typically induce a more pronounced complement response^53^. Despite this, *P. aeruginosa* from CF sputum can sometimes evade the activated complement system^54^. In order to avoid a complement attack, *P. aeruginosa* attracts Factor H (FH) to its surface. This study finds that Factor H-related protein 3 (FHR-3) can outcompete FH, binding to EF-Tu on the bacterial surface. Elevated FHR-3 levels enhance complement activation, increasing opsonization and bacterial killing, highlighting FHR-3’s role in countering *P. aeruginosa* immune evasion^55^.

### Role of virulence factors in innate immune response

Pyocyanin and other virulence markers produced by *P. aeruginosa* in biofilms have the potential to adversely affect cells and interfere with immune system responses in CF lungs^56^. Pyocyanin impairs ciliary function and promotes mucin overproduction, creating a cycle that favors biofilm formation and dysregulated immunity^57^. Although the biofilm in CF lungs should be vulnerable to antimicrobial peptides from pulmonary epithelial cells and neutrophils, the acidic conditions in CF lungs lower the potency of these polypeptides^58,59^. Salt imbalances and other factors in CF lungs can reduce the effectiveness of antimicrobial peptides^60^. These factors involve the breakdown of peptides by both bacterial and host-derived proteases, as well as the suppression of peptide activity through their attachment to complexes formed with F-actin, mucins, LPS and DNA released from the host^61,62^.

## Innate immune reactions to *P. aeruginosa* in respiratory diseases

Chronic obstructive pulmonary disease (COPD) is a long-term, worsening respiratory disorder marked by restricted airflow, inflammation of the airways, lung tissue damage (emphysema), and increased mucus secretion caused by narrowed air passages. The condition is mainly triggered by prolonged exposure to harmful substances such as cigarette smoke and environmental pollutants, as well as influenced by genetic predispositions and other environmental contributors^63^. Although *P. aeruginosa* infections are less frequent in COPD compared to CF or non-CF bronchiectasis (NCFB), their prevalence rises from 4% to 13% in individuals with severe airway obstruction^64,65^. Given that COPD affects significantly more individuals than CF, the impact of *P. aeruginosa* infections remains a critical concern, especially with their role in exacerbating disease severity^66^. Exacerbations of COPD caused by *P. aeruginosa* tend to be more severe, leading to higher hospitalization rates and increased mortality compared to other pathogens^63^. *P. aeruginosa* adapts to persistent infections by going through clonal diversification and acquiring characteristics including decreased protease activity, biofilm development, hypermutability, resistance to antibiotics, and decreased motility. These changes promote bacterial persistence, making infections more difficult to treat and contributing to chronic disease progression^67,68^. The innate immune components, like neutrophils, macrophages, NK cells, PRRs, alginate, *Pel*, *Psl*, the complement system, and various virulence factors, are largely similar across CF, COPD, and VAP infections. In COPD exacerbations, the innate immunological reaction to *P. aeruginosa* biofilms includes a complicated interaction of evasion strategies, inflammation, and immune recognition^69^. Bacteria within biofilms release components like LPS, flagellin, and eDNA, which trigger PRRs including TLRs and NOD-like receptors (NLRs)—found on epithelium, macrophages, and neutrophils^70^. TLR4 detects LPS, TLR5 recognizes flagellin, and TLR9 senses bacterial DNA, initiating an inflammatory cascade^71^. Cytokines promoting inflammation, including IL-8, TNF-α, and IL-1β, are released as a result, drawing in neutrophils, monocytes, and NK cells. IL-17 from innate lymphoid cells (ILCs) further amplifies neutrophilic infiltration, intensifying inflammation^72^. As the main responders, neutrophils produce ROS and discharge antimicrobial peptides like cathelicidins and defensins to break down the biofilm matrix. Additionally, they produce neutrophil extracellular traps (NETs), which are made of DNA and enzymes like neutrophil elastase. While these structures help trap and control bacterial spread, they may also contribute to the deterioration of lung tissue^73^. Alveolar macrophages (AMs) attempt to clear planktonic bacteria through phagocytosis, though biofilm-associated bacteria display increased resistance. These macrophages also produce matrix metalloproteinase-9 (MMP-9), which degrades biofilm components but may exacerbate tissue remodeling and damage^74^. Persistent biofilms sustain chronic immune activation, resulting in a cytokine storm dominated by IL-1β, IL-6 and IL-8 driving tissue destruction and fibrosis, which further compromises pulmonary function in COPD patients^75^. Recent data indicate that *P. aeruginosa* biofilms in the airways of individuals with CF are strong inducers of M1 macrophage polarization, which leads to an environment that is hyperinflammatory^76^. Notably, while these biofilm-rich environments elevate IL-8 and neutrophil elastase levels, sputum IL-6 concentrations remain paradoxically low, suggesting a distinct CF-specific inflammatory signature. This divergence supports the concept that biofilms can induce pro-inflammatory immune phenotypes while modulating specific cytokine outputs like IL-6^77^.

Ventilator-associated pneumonia (VAP) is a respiratory condition that occurs shortly after an individual has been on a ventilator for more than 48 hours. It’s one of the most prevalent infections picked up in the ICU, affecting ~20 to 36% of seriously ill patients^78^. A key factor in VAP development is bacterial biofilm formation on endotracheal tubes (ETTs), which occurs in 95% of ventilated patients. These biofilms serve as reservoirs for pathogens that can dislodge, travel into the lower airways, and trigger infection^79^. The longer an individual is on mechanical breathing, the higher probability of biofilm development, which is thought to occur before VAP and starts within hours after intubation^80^. AMs, among the most prevalent immune cells in the lower part of the respiratory system, play a vital role in maintaining lung homeostasis and reacting to infections^81^. Through NF-κB-driven cytokine production, including TNF-α, IL-1α, IL-1β, IL-6, and IL-8, they identify and engulf pathogens and coordinate the innate immune response, attracting neutrophils to fight infection. AMs are antigen-presenting cells that also trigger adaptive immunity, which causes congestion, capillary leakage, and inflammation of the lungs. When local defences are overwhelmed, both AMs and epithelial cells amplify cytokine signaling to drive neutrophil migration into lung airspaces^82^. When bacterial infections cause VAP, neutrophils are drawn to the lungs in large numbers. There, they use antimicrobial defences such as phagocytosis, NETs degranulation and the generation of ROS. Histones and peptides with antimicrobial properties present in NETs help eradicate infections, but they may also encourage long-term inflammation^83,84^. Research has shown that VAP patients had higher blood CRP and IL-6 levels as well as enhanced pulmonary biomarkers such as MMP-8, MMP-9, IL-1β, IL-8, and human neutrophil elastase (HNE). While neutrophils are essential for pathogen clearance, excessive NET formation can exacerbate lung injury and elevate mortality risk by perpetuating localized inflammation^85^.

## Innate immune reactions to *P. aeruginosa* in chronic wound infections

Recent studies on persistent wound infections have shed fresh light on immunological reactions to *P. aeruginosa* biofilms, however the majority of our knowledge has come from researching CF lung infections. The rise in persistent wounds, often linked to obesity and lifestyle diseases, has become a significant concern. In chronic wounds, the immune response is characterised by prolonged inflammation, leading to ongoing oxidative damage, fibroblast ageing, and disrupted growth factors essential for healing. This complex pathology also involves low cell growth activity, high protease levels with insufficient inhibitors, shifts in the microbiome, and the nature of the initial injury and invading pathogen. There is increasing evidence that microbial biofilms are important in affecting the patient’s immunological response and slowing the healing process in these wounds.

**Fig. 2:**
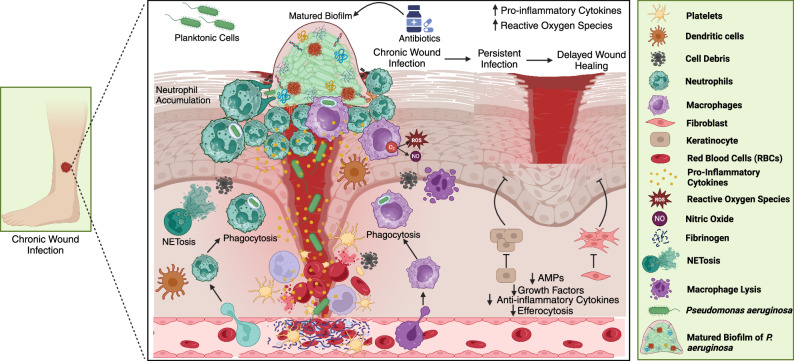
Innate immune response to biofilms in chronic wound infections. This figure illustrates the interplay between the innate immune system and *Pseudomonas aeruginosa* biofilms in chronic wound infections. The wound site initially accumulates planktonic bacterial cells that evolve into mature biofilms, which are resistant to antibiotic treatment. Neutrophils are recruited to the site and initiate NETosis, releasing neutrophil extracellular traps (NETs) to capture biofilm-associated bacteria. Macrophages also engage in phagocytosis to clear pathogens, while dendritic cells, fibroblasts, and keratinocytes contribute to the immune defence and tissue repair. Despite these responses, the biofilm creates a persistent infection characterized by increased levels of reactive oxygen species (ROS) and nitric oxide (NO), as well as pro-inflammatory cytokines. These factors inhibit efficient wound healing, further delaying recovery. The prolonged inflammation causes tissue damage and impairs the production of antimicrobial peptides (AMPs), growth factors, and anti-inflammatory cytokines, leading to ineffective efferocytosis, macrophage lysis, and continued infection persistence. This cycle results in delayed wound closure, underscoring the difficulty in eradicating biofilm-based infections in chronic wounds.

*P. aeruginosa* has a well-established effect on wound persistent nature in both clinical and experimental settings^86,87^. It is now commonly acknowledged that biofilms are the main source of chronic infections, causing persistent pathology regardless of ongoing host immune system reactions and antibacterial therapies^88^. Rhamnolipids, which are part of *P. aeruginosa* biofilms, are thought to be essential for maintaining infections because they cause cellular necrosis and kill neutrophils^11^. Innate immune response to biofilms in chronic wound infections is explained in Fig. 2.

Endogenous antimicrobial peptides (AMPs), which are evolutionarily ancient, are essential to the skin’s innate defence against infection^89^. In reaction to bacterial infection, wound closure, trauma, prolonged inflammatory conditions, keratinocytes, granulocytes, and macrophages release these peptides, which also have regenerative qualities^90^. Because of their dualistic nature, AMPs can attach to microbial membranes, creating pores that lead to bacterial lysis^91^. The host defence protein S100A8/A9, classified among the alarmins, performs multiple functions and is found in actively healing wounds in both human and murine models^92^. However, S100A8/A9 is lacking in persistent, infected varicose ulcers in individuals, most likely due to a disturbed local immune response, which may delay wound healing^93^.

Two mice strains have been used to create a chronic wound model in order to examine the relationship among biofilms and the body’s immune reaction: BALB/c, which is more susceptible *P. aeruginosa* infection, and C3H/HeN, which is comparatively resistant^20^. BALB/c mice exhibit a Th2-biased immune system reaction, whereas C3H/HeN rodent generally mount a Th1-skewed immune reaction against infections such *Leishmania major* and *Candida* species. This Th response difference significantly affects mortality rates and infection clearance^94^. During the first five days of infection in a persistent wound model, C3H/HeN rodents showed a reduced local IL-1β inflammatory reaction against *P. aeruginosa* biofilm in comparison to BALB/c mice^20^. Furthermore, new research suggests that *P. aeruginosa* biofilm might weaken the native neutrophil reaction in several mouse wounds, which could compromise infection management. Other investigations have confirmed the slow recovery linked to the BALB/c genotype, which makes this strain a perfect model for studies on wound repair^95^. In this scenario, examining the gradual recovery of *P. aeruginosa* biofilm-mediated infection wounds in C3H/HeN and BALB/c mice in conjunction with S100A8/A9 gene expression may provide crucial insight into the significance of S100A8/A9 in the process of wound repair. Using a murine wound model, a recent study evaluated the effectiveness of a new HOCl-producing electrochemical bandage in decreasing *P. aeruginosa* biofilms, obtaining significant CFU reductions while preserving normal wound healing. Compared to control treatments, polarized e-bandages significantly lowered biofilm load and purulence, positioning this approach as a promising, antibiotic-free option for wound infection management^96^.

## Adaptive immune mechanisms in response to *P. aeruginosa* biofilm infections

The ability of the adaptive immunological system to differentiate between the host’s proteins and foreign substances is essential for directing lymphocyte and antibody-mediated responses against infections rather than the host^97^. Unlike the innate immune system, which offers a more generalized response, the adaptive immune system reaction is extremely specific. Upon re-exposure to a previously encountered microbe, it rapidly expands antigen-specific effector and memory cells by up to 1000-fold, providing immunity against future infections^98^. In contrast to innate immunity, this secondary reaction is far more rapid, more powerful, and more specific than the primary response^99^.

**Fig. 3:**
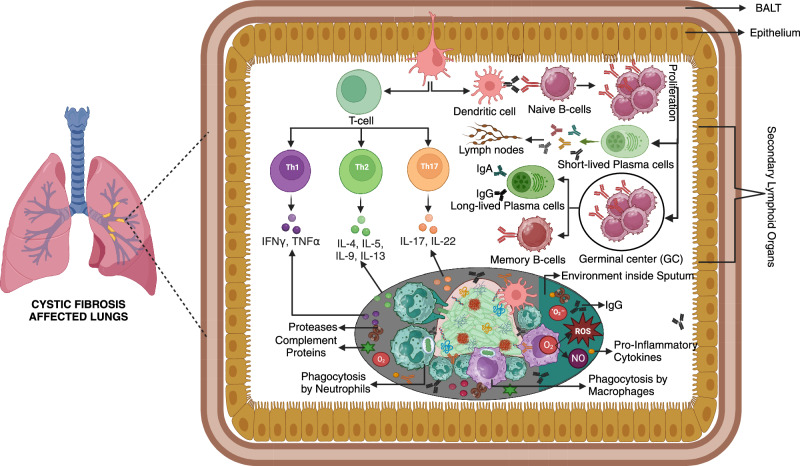
Adaptive immune response to *Pseudomonas aeruginosa* biofilms in cystic fibrosis lungs. This figure illustrates the adaptive immune mechanisms in response to *Pseudomonas aeruginosa* biofilms within the cystic fibrosis airway. The biofilm environment is rich in extracellular polymeric substances, providing protection against immune attacks. Dendritic cells capture biofilm-associated antigens and activate naive T-cells, differentiating them into Th1, Th2, and Th17 subsets. Th1 cells release IFNγ and TNFα, enhancing phagocytosis by neutrophils, while Th2 cells secrete IL-4, IL-5, IL-9, and IL-13, promoting B-cell responses. Th17 cells release IL-17 and IL-22, driving inflammation. Activated B cells differentiate into short-lived plasma cells producing IgA and IgG, or migrate to germinal centres (GC) to form long-lived plasma cells and memory B cells, providing prolonged immune defence. Despite these efforts, the biofilm structure limits immune cell access, while reactive oxygen species (ROS) and nitric oxide (NO) are generated to combat infection. Pro-inflammatory cytokines and complement proteins further contribute to the immune response, though the biofilm’s protective matrix often leads to persistent infections.

The pathogen can frequently be eliminated during acute infections by the integrated efforts of the innate and adaptive immunological systems. But this pathogen is not completely eliminated in persistent infections, including those linked to *P. aeruginosa* biofilms^100^. The ability of infections linked to biofilms is largely determined by the cooperation among the innate and adaptive immunological processes, wherein the adaptive reaction is initially delayed^6,101^. Figure 3 describes the adaptive immune response to *P. aeruginosa* biofilms in the lungs of patients with cystic fibrosis. Table 2 summarizes the adaptive immune system components and their function in *P. aeruginosa* biofilm infections. Studies indicate that CFTR modulators may influence lung inflammation and immune responses, with in vitro evidence showing reduced inflammatory activity in CFTR-deficient cells. For instance, treatment of CFTRF508del epithelial cells with lumacaftor/ivacaftor resulted in lower CXCL8 production and improved tissue repair upon *P. aeruginosa* exposure, suggesting a modulatory effect on inflammatory responses^102^.

**Table 2:** Therapeutic strategies for targeting *Pseudomonas aeruginosa* biofilms

Therapy type	Target/mechanism	Key effects	Study results and findings	References
IgY antibodies	IgY antibodies derived from chicken egg yolk target bacterial flagella, hindering adhesion to host cells.	Inhibits bacterial colonization and enhances immune cell-mediated clearance of bacteria.	Reduced lung colonization in animal models; effective in preventing chronic colonization in cystic fibrosis (CF) patients.	176 196 221
S100A8/A9 proteins	Recombinant proteins modulate host immunity, reduce biofilm formation, and enhance antibiotic efficacy.	Promotes bacterial killing and modulates wound healing processes in biofilm-infected wounds.	Reduced bacterial load in murine models of wound infection; promising therapeutic effects with systemic antibiotics.	165
3 C patch therapy	Autologous fibrin-rich patches containing leukocytes/platelets enhance wound healing and immune response.	Accelerates healing of chronic wounds and enhances leukocyte activity (phagocytosis and respiratory burst).	Open studies show accelerated wound closure in patients with non-healing wounds of various causes.	^167,168^
KB001-A (monoclonal Ab)	Blocks *P. aeruginosa* Type III Secretion System (T3SS) by targeting PcrV protein, reducing bacterial virulence.	Decreases bacterial virulence and inflammation by reducing the activity of T3SS.	Phase I/II studies showed safety, with reduced inflammatory markers (e.g., IL-8) in CF patients.	^174^ ^175^
Panobacumab (mAb)	Human IgM monoclonal antibody targets *P. aeruginosa* lipopolysaccharide (LPS) to prevent colonization.	Inhibits biofilm formation and decreases recurrence rates of bacterial infections.	Phase IIa trials showed reduced recurrence of nosocomial pneumonia and enhanced patient recovery.	^177^ ^178^ ^179^
Recombinant flagellin vaccine	Vaccine developed from flagellar antigens stimulates humoral immune responses, reducing bacterial load.	Enhances the production of specific antibodies (anti-flagellin), reducing bacterial colonization and inflammation.	Preclinical studies in murine models showed a significant reduction in lung inflammation and bacterial load.	^201^
**Trivalent DNA vaccine**	Incorporates genes (oprL, oprF, flgE) from *P. aeruginosa* to stimulate both cellular and humoral immunity.	Induces a strong immune response, producing cytokines (IFN-γ, IL-2, IL-4) and enhancing lymphocyte activity.	Protective efficacy demonstrated in animal models; further validation required in clinical trials.	^186^
Epitope-based Vaccines	Designed using bioinformatics tools to identify and target key bacterial epitopes for B and T cell response.	Triggers both cellular and humoral immune responses targeting multiple antigens, reducing bacterial infections.	In silico studies identified potential epitopes; in vivo and in vitro studies are still needed to confirm efficacy.	^191^
PHA-coated bead vaccine	Biodegradable beads conjugated with outer membrane proteins (e.g., AlgE, OprF) trigger T1-type immunity.	Induces robust immune responses (IFN-γ, IgG2c), promoting opsonophagocytic killing of *P. aeruginosa*.	Preclinical murine studies showed effective immune responses and bacterial clearance.	^190^
Glycoconjugate vaccine	Conjugates LPS or OPS antigens to carrier proteins, enhancing immunogenicity and protection against biofilms.	Stimulates the production of antibodies (IgG, IgA, IgM) specific to *P. aeruginosa* and reduces biofilm formation.	Animal models demonstrated protective immunity; ongoing development for human trials.	^188^ ^189^

This table provides a concise overview of therapies aimed at combating *Pseudomonas aeruginosa* biofilms, highlighting their targets, effects, and study outcomes. IgY antibodies, derived from egg yolk, target bacterial flagella, reducing adhesion and colonization, proving effective in animal models and cystic fibrosis patients. S100A8/A9 proteins modulate the immune response, decrease biofilm formation, and boost antibiotic activity in wound infections. 3 C Patch Therapy aids chronic wound healing through immune enhancement. KB001-A blocks T3SS, reducing bacterial virulence and inflammation. Panobacumab targets LPS, preventing biofilm formation and infection recurrence. Flagellin and trivalent DNA vaccines induce immune responses to reduce bacterial load, with ongoing validation. Epitope-based vaccines target bacterial epitopes but need further testing. PHA-coated beads and glycoconjugate vaccines stimulate immune responses and have shown efficacy in animal models.

## Adaptive immune response in CF

### Role of DCs in adaptive immune response

Dendritic cells (DCs) are the primary force behind the activation of the adaptive immunological reaction. After the initial encounter with a pathogen, they cooperate with macrophages (Mφ) to start the immune response^103^. Peripheral tissues contain immature DCs, which are particularly effective at collecting antigens. They are more prevalent in places that are exposed to infections, like secondary lymphoid tissues (SLTs) and mucosal surfaces^104^. Upon capturing antigens and being influenced by inflammatory cytokines, these immature DCs mature into cells specialized in processing and presenting antigens^105^. Due to their unique capacity to stimulate naïve T-cells into unique parts, such as Th1, Th2, and Th17 cells, DCs serve as the crucial connection within the innate and adaptive immunological systems, making the process of maturation crucial^106^. According to ref. ^107^, isolating DCs is particularly challenging in human studies due to their limited presence in tissues. However, we found that pulmonary DCs are involved early in the infection process in our experiments utilising a persistent *P. aeruginosa* pulmonary infection model. Especially, pulmonary DCs were seen as early as two days from the onset of the infection; a rise in DCs in the local lymph nodes wasn’t seen until day seven. The extent of expression of CD80 and CD86, which indicate the activation of pulmonary DCs, rose gradually throughout the duration of the 10-day observation period. By the 10th day, the DCs’ activity in the lymph nodes had diminished. Surprisingly, cytokines that promote inflammation IL-6 as well as IL-12 spiked between day 2 and 3, and subsequent production of IL-10 increased by day seven. The cytokine production patterns of DCs from the respiratory and lymphatic systems largely resembled each other. This pattern indicates that DCs serve as a vital role in controlling the creation of adaptive immunological responses, which are impacted by the innate immunological activity in the environment^107^. Further study has demonstrated that *P. aeruginosa* quorum-sensing components may hinder the synthesis of IL-12 in murine DCs while preserving the release of IL-10. Additionally, QS-exposed DCs showed reduced antigen-specific proliferation of T cells, indicating that *P. aeruginosa* QS signals may impair T-cell activation, therefore increasing the resilience of *P. aeruginosa* biofilms^108,109^. Recent findings on the effects of GM-CSF and G-CSF on DCs have led to the idea that, in addition to drawing PMNs from the bone marrow (BM), elevated G-CSF levels may also influence the DC response in CF patients with prolonged *P. aeruginosa* infection^110^. Researchers found a significant correlation between the GM-CSF/G-CSF proportion and IFN-γ reaction, which was also associated with enhanced lung function^111^. On the other hand, IL-3 and IFN-γ responses showed an inverse correlation. DCs play an integral role in the body’s reaction to biofilm infections, making them a promising therapeutic target^6^.

When chronic infections caused by biofilm are present, the cooperation of the innate and adaptive immunological systems might lead to tissue damage^112^. Inflammatory cells release oxidative radicals and enzymes, which, combined with bacterial virulence factors like elastases and proteases, degrade crucial immune cell surface molecules, further impairing the host’s anti-biofilm defences^6^. Interestingly, despite the presence of antibodies against several bacterial virulence factors in chronic biofilm infections, these antibodies do not appear to improve the outcome. Instead, they may contribute to pathology by forming immune complexes that activate the complement system and promote tissue-damaging inflammation^113^.

### Role of immunoglobulins in adaptive immune response

The development of mucosal antibodies, particularly secretory IgA (sIgA), is a noteworthy feature of the body’s adaptive immune reaction in *P. aeruginosa* pulmonary infections. Unlike IgG, which is part of the systemic immune response and infiltrates mucosal surfaces through inflamed epithelium, sIgA is specifically produced at mucosal sites^114^. It plays a protective role by binding to pathogens and their PAMPs, preventing complement activation and opsonization. IgG is more common in the lower airways and is linked to pulmonary inflammation, but sIgA is frequently observed in the sinus cavities of CF individuals and is connected with chronic sinusitis. Additionally, early identification of *P. aeruginosa* in CF patients’ lower airways has been correlated with the presence of sIgA^115^.

### Role of T cells in adaptive immune response

*P. aeruginosa* induced persistent lung infections in CF patients best illustrating the connection between biofilm infections and the immune system’s response^108^. Standard treatment now includes early aggressive antibiotic therapy, ongoing maintenance between exacerbations, and planned intravenous antibiotics^116^. The way these pulmonary infections develop in CF patients differs despite these treatments. Most patients experience a poor outcome with a sharp rise in antibody levels, while a smaller group shows a modest immune response and enjoys better outcomes^117^. Interestingly, more intensive antibiotic treatments tend to yield better results, which are linked to lower antibody responses^118^. A Th1/Th2 cytokine contradiction has been identified in CF individuals who have persistent infections based on studies of cytokine generation by reactivated peripheral blood mononuclear cells (PBMCs) and subsequently by unspecific activated T cells^119,120^. These individuals have a Th2-dominated cytokine profile, characterized by boosted IL-4 production (combined with IL-5 as well as IL-10) and decreased IFN-g^121^. An analogous cytokine pattern was seen in BAL fluid from certain groups of CF patients^122,123^. Surprisingly, the production of IFN-g by PBMCs was linked to enhanced lung function, indicating that IFN-g may have a positive effect^119^. This observation is supported by studies in inbred mouse strains, where the comparatively resilient C3H/HeN mice with persistent *P. aeruginosa* lung infections displayed significant pulmonary IFN-g levels^94,124^. Re-infection of normally more sensitive BALB/c mice led to a respiratory Th1 reaction that resembled that of C3H/HeN mice, simulating the progression of an initial infection in the second group^124^.

The specific reasons for enhanced results in those suffering from CF with recurrent *P. aeruginosa* pulmonary infections remain unresolved, especially because a Th1 response is generally more efficient against intracellular organisms. Interestingly, one theory claims that AMs phagocytose apoptotic polymorphonuclear neutrophils (PMNs) before they necrotize and worsen inflammation^125^. Another potential mechanism could be the reduction of IL-8, a key chemoattractant for PMNs^126^. A less prominent Th2 response may result in decreased activation of B and plasma cells, immunological complex development, lowering antibody production and eventual damage to tissues^127^.

Several subsets of T cells have also been found, including Th17 cells, which generate IL-17 and occasionally IL-22^128,129^. Th17 cells, produced by TGF-b 129, might have an important role in CF because IL-17 stimulates the synthesis of G-CSF, a PMN mobilizer, and IL-8, a chemoattractant. Thus, Th17 cells may have a role in the lung pathophysiology connected to persistent infections caused by *P. aeruginosa*^130,131^. In contrast to individuals with CF without persistent *P. aeruginosa* infections, sputum from stable CF patients and those with persistent infections had larger amounts of IL-17 and IL-23. This rise, however, was not detected in CF patients diagnosed with *S. aureus*^130^. According to reports, CF patients have a much lower percentage of peripheral Th17 cells, which may indicate that these cells are better suited to the lungs, where they can worsen inflammation^132^. Research on children with CF has revealed that symptomatic individuals had greater quantities of IL-17A along with the Th2-related cytokines IL-5 and IL-13^131^. In contrast, no such association was detected for Th1-associated cytokines, showing a possible relationship among the Th2 and Th17 groups in CF^131^. Although a Th2-Th17 pathway could expose CF patients to *P. aeruginosa* pulmonary infections, this connection has yet to be entirely understood^131,133^.

CF patients have recently been found to contain T-cell inhibitory neutrophil myeloid-derived suppressor cells (MDSCs)^134,135^. The occurrence of neutrophil MDSCs in the peripheral circulation was unexpectedly linked to enhanced lung function in CF, which contradicted predictions^134^. Downregulation of the prominent and damaging Th2 and Th17 reaction axes might contribute to this finding.

T cell groups, including the regulatory T cells and Th22 cells, have received little attention in the setting of biofilm infections. However, research indicates that CF patients could have fewer of these immune system cells and their activity may be reduced, which might result in elevated concentrations of IL-17 and IL-8^133,135^.

### Role of B cells in adaptive immune response

B cells originate from hematopoietic stem cells, with their development progressing through various stages, beginning in the BM^136^. The cycle of maturation begins in the BM and proceeds when B cells move to SLTs such as the spleen and lymph nodes^137^. In the SLTs, B cells undergo activation and differentiation, driven by changes in gene expression within germinal centres (GCs). GCs are transitory complexes found within SLTs that allow B cells that create high-affinity antibodies to mature, get activated, and eventually grow into either blood cells or as memory B cells. During growth and development, B cells reorganise gene segments of immunoglobulin (Ig) chains, resulting in the creation of a mature B-cell receptor (BCR) on the cell membrane that can interact with antigens. Interactions with BM stromal cells, which supply essential signals including chemokines, integrins, growth factors, and cytokines, are essential for this maturation. After leaving the BM, embryonic B cells grow in SLTs with the help of transcriptional factors like NOTCH2 and BTK, BCR signals, and the B-cell activating factor (BAFF)^138^.

In *P. aeruginosa*-infected CF patients, high numbers of B cells have been observed, with levels decreasing during treatment for pulmonary exacerbations. These patients also show high levels of immunoglobulins, particularly IgA, in the airway surface liquid (ASL). However, this immune response is inadequate to eliminate pathogens, stop chronic infection, or reduce the spread of new strains^139^ Moreover, a greater spontaneous production of biofilm-forming cells, which indicates B-cell transition into blood cells, has been seen among individuals with CF. Interestingly, these individuals show decreased B-cell development in reaction to multifaceted activation in vitro, which can’t be explained by the presence of suppressor cells or greater T-cell suppression^140^. According to recent research, *P. aeruginosa* infection is associated with higher quantities of BAFF and other B-cell chemoattractants such as CCL21, CXCL19, and CCL13 in a CF mouse model. The BAL of pediatric patients with CF has also been reported to have enhanced BAFF levels, irrespective of *P. aeruginosa* infection. This implies that the lung environment associated with CF stimulates B-cell survival, differentiation, and antibody generation^141^. But the fact that the *P. aeruginosa* infection continues to persist suggests that this B-cell response might not be functioning. Despite these results, more investigation is required to ascertain if CF affects B-cell production, regulation, and proliferation^142^.

## Adaptive immune response in respiratory diseases

An important function of DCs is to narrow the gap between innate and adaptive immunity. Cigarette smoke promotes DC accumulation and chemokine release, contributing to COPD progression^143,144^. COPD patients exhibit increased pulmonary immature DCs, while mature DCs—marked by CD83+ and DC-LAMP(+)—decline in active smokers but recover after smoking cessation. Patients with COPD consistently show lower levels of mature DCs than non-smokers and former smokers without COPD. Activated DCs drive Th1 and Th17 responses, CD8+ cytotoxicity, and B-cell activation, leading to lymphoid follicle formation in chronic inflammation^145^. Additionally, COPD-associated DCs show elevated CD80/CD86 expression and increased IFN-α secretion. In COPD, adaptive immune reactions are induced by cigarette smoke-derived antigens, pathogens, and autoantigens, which involve B cell-based antibody formation Th1, CD8+ cytotoxic T cells, and Th17 CD4+ cells^146^. Individuals with COPD have an increase of CD8+ T lymphocytes in the bronchial lining and pulmonary lumen, as well as a reduction CD4+/CD8+ ratio in the paratracheal lymph nodes. These cells contribute to disease progression through IFN-γ production and cytotoxic activity via perforin and granzyme B, leading to cell death and emphysema. Additionally, CD8+/CD28− T cells, elevated in COPD, show increased IFN-γ, granzyme, and perforin expression under cigarette smoke exposure and reduced glucocorticoid receptor levels, contributing to steroid resistance. Targeting CD137 on these cells may help suppress inflammatory mediators, offering a potential therapeutic strategy^147^. CD4 + T cells have a substantial function in maintaining inflammation by encouraging CD8 + T cell survival and activating B cells to produce antibodies. In COPD, these cells may drive autoimmune responses, contributing to disease progression. They potentially enhance B cell generation of IgG antigen-specific antibodies. Approximately 70% of people with COPD carry IgG autoantibodies against epithelial cells^147^.

In VAP, T cells and B cells play pivotal roles in modulating the immune system response. Recent research indicates the importance of Th17 cells, a division of T helper cells, in giving protection against VAP. Patients with VAP exhibit a notably lower proportion of Th17 cells in their alveolar fluid compared to those without VAP. This reduction is associated with decreased levels of interleukin-17A (IL-17A), a cytokine instrumental in recruiting neutrophils to infection sites, thereby enhancing bacterial clearance^148^. Research indicates that mechanical ventilation can suppress T-helper 17 (Th17) cytokines, such as IL-22, IL-17A, and IL-17F, which are crucial for mucosal immunity and defence against bacterial infections. This suppression may increase susceptibility to VAP^149^. Studies have shown that patients with VAP exhibit a decrease in CD3⁺/CD4⁺ T-helper lymphocytes and increased apoptosis of CD14⁺ monocytes, leading to immunoparalysis and impaired immune defence^150^. The way the immune system responds to VAP depends heavily on B cells. Recent research has underlined the significance of lung-resident memory B cells (BRM cells) in providing localized immunity. These BRM cells, identified in both mice and humans, persist in the lungs after initial infections and are poised to secrete antibodies upon re-exposure to pathogens, thereby enhancing bacterial clearance^151^. This highlights a significant research gap; therefore, additional studies are needed to better characterize the adaptive immunological response in COPD and VAP to fully understand the similarities and differences compared to CF.

## Adaptive immune response in wound infections

In wound infections caused by *P. aeruginosa* biofilms, the adaptive immune response is significantly challenged. DCs and macrophages, which are pivotal for antigen presentation and T-cell activation, often exhibit impaired function due to biofilm-induced alterations. This dysfunction results in inadequate stimulation of CD4+ and CD8 + T cells, which are critical players in orchestrating effective immunological responses. The extracellular polymeric material (EPS) in the biofilm serves as a physical barrier that prevents B-cell-produced antibodies from penetrating, decreasing their ability to neutralize the bacteria^6^. Moreover, *P. aeruginosa* biofilms can modulate cytokine production, skewing the immune response towards a Th2 profile, which is less effective in bacterial clearance. This modulation not only facilitates persistent infection but also contributes to chronic inflammation and tissue damage^121^. Recent investigations have shown that the long-term persistence of biofilm infections causes ongoing stimulation of the adaptive immune system, leading to immunological fatigue and reduced response over time. Despite advances in immunological research, complete data on how the immune system reacts to *P. aeruginosa* biofilms in wound infections is still lacking. Further investigation is required to comprehend the immune system mechanisms and find effective treatments for these chronic infections.

## Immune evasion strategies of *P. aeruginosa*

*P. aeruginosa* has advanced strategies for avoiding intracellular death after being swallowed by immune cells like macrophages and neutrophils. Instead of being efficiently degraded within the phagolysosome, the bacterium can manipulate host cell processes to resist oxidative and enzymatic attacks. It accomplishes this by interacting with phagosomal maturation and using type III secretion system (T3SS) effectors such as ExoS and ExoT to alter cytoskeletal integrity and cause apoptosis^152^. Another key mechanism involves the secretion of elastase, an enzyme that degrades pulmonary surfactant protein-A (SP-A), a crucial component in the host’s defense system. By breaking down SP-A, *P. aeruginosa* reduces opsonization, thereby diminishing its recognition and uptake by phagocytes^153^. Upon engulfment by macrophages, the bacterium exhibits a remarkable ability to persist rather than being eradicated. Persister cells of *P. aeruginosa* are internalised at a far slower pace than actively developing cells, and once inside macrophages, they remain alive rather than being destroyed by phagosomal destruction^154^. Once internalized, persister cells evade destruction and, unlike their actively growing counterparts that drive a pro-inflammatory M1 macrophage response, they initially promote an M2-like polarization characterized by elevated IL-10 and moderate levels of CXCL-8, IL-6, and TNF-α. However, as the infection progresses and persister cells reawaken, macrophage polarization shifts toward a more inflammatory M1 state, reinforcing the immune response^154^. Another critical mechanism of immune evasion is the bacterium’s ability to resist complement-mediated killing. Although C3b opsonization occurs similarly to typical vegetative cells, persister cells have lower levels of C5b, the major activator of the membrane attack complex (MAC), which prevents complement-driven lysis^154^. The biofilm matrix of *P. aeruginosa* effectively conceals LPS from immune recognition via TLRr 4 (TLR4). In TLR4-deficient models, unprotected LPS exposure induced a stronger sickness response compared to EPS-coated bacteria. Moreover, a lung-to-brain communication pathway was identified, where TLR4-expressing TRPV1+ sensory neurons activated stress responses in the hypothalamus rather than direct inflammation. These findings reveal how biofilm-mediated immune evasion and neuroimmune signalling shape disease progression and highlight novel therapeutic targets within the lung–brain axis^155,156^.

## Innovative therapeutic approaches for combating *P. aeruginosa* biofilm infections

Passive immunisation therapy is the use of pre-produced immune system antibodies or immunoglobulins to combat a variety of infectious diseases. A promising approach within this domain is the use of avian IgY immunoglobulins, derived from egg yolk, as a substitute for traditional antibiotics for treating *P. aeruginosa*. IgY, the most prevalent antibody found in chicken serum and a birds equivalent to mammalian IgG^157^, builds up in the egg yolk, supplying the baby with vital humoral immunity. The egg yolk can contain large quantities of particular IgY antibodies if hens are highly immunised with certain antigens. The application of IgY to *P. aeruginosa* provided promising results^158^. In-vitro investigations showed that these antibodies bind strongly to bacterial flagella, affecting the bacteria’s adherence to epithelial cells and perhaps preventing respiratory tract colonization^159^. Furthermore^160^, has discovered that anti-*P. aeruginosa* IgY increases the respiratory burst of PMNs and bacterial death of *P. aeruginosa* in vitro. The antibodies appear to stimulate microbes’ accumulation, leading to encapsulation and enhanced surface hydrophobicity, which in turn facilitates phagocytosis that is independent of Fc receptors^161^. Anti-*P. aeruginosa* IgY treatment significantly decreased the pulmonary bacterial load by two logs and decreased airway inflammation in mice treated with anti-specific IgY, which confirmed these in vitro results in in vivo experiments using an acute murine pneumonia model^162^. This suggests that pathogen-specific IgY might not only enhance PMN-mediated phagocytosis but also reduce airway colonization in CF patients. Furthermore, Igy may operate synergistically with anti-pseudomonal medicines^163^, as proven by clinical research that found good outcomes when non-chronically infected CF patients received oral preventive immunotherapy with anti-*P. aeruginosa* IgY^164^.

In parallel, recombinant S100A8/A9 proteins have also shown potential as therapeutic agents. Our team demonstrated that applying recombinant S100A8/A9 locally for four days, alongside systemic ciprofloxacin, significantly reduced the bacterial burden in biofilm-infected wounds on BALB/c mice^165^. Interestingly, this effect appeared to be dependent on host cell interactions, as no synergistic effect was observed between S100A8/A9 and ciprofloxacin in vitro. Ongoing research aims to further comprehend the complicated function of S100A8/A9 in the pathological process of biofilm-infected wounds, especially as both human and animal studies indicate an impaired or inadequate S100A8/A9 response in non-healing wounds^165^. The creation of a gelatin nanoparticle-based vaccine containing a hybrid protein of *P. aeruginosa* ExoS and OprI proteins shows potential effectiveness, as evidenced by the generation of significant systemic and mucosal immune system reactions in mice. With high encapsulation efficiency, sustained antigen release, and effective lung protection against respiratory infections, this CP-GNP nano vaccine could represent a valuable preventative strategy against *P. aeruginosa*^166^.

In the scenario of treating wounds that are not healing where the host’s anti-biofilm response is inadequate, autologous fibrin-rich patches incorporating platelets and leukocytes have emerged as a viable treatment. One such treatment includes generating a “3 C patch” by centrifuging the individual’s complete blood using a customized device^167^, and then placing the patch onto the persistent wound^168^. An open trial on individuals with persistent wounds from varied sources found that the majority achieved quicker healing with the 3 C patches^169^. This impact is probably triggered by platelet-mediated release of growth factors as well as cytokines such as PDGF-bb^167^. Additionally, significant PMN activity, including respiratory burst, phagocytosis, and anti-biofilm effects, was observed within the 3 C patches^168^. Photodynamic (PDT) and photothermal (PTT) therapies are now recognized as viable treatments for biofilm infections, with PTT increasing antibiotic penetration while decreasing resistance. Although NIR light can achieve significant biofilm reduction, high irradiation levels and photosensitizer concentrations risk tissue damage and inflammation, highlighting the need for optimized therapeutic parameters^170^. A study examined the innate immune activation and metabolic adaptations in *P. aeruginosa* PAO1 mutants resistant to LPS- and T4P-targeting phages, revealing diverse genomic modifications. Mutants with large gene deletions showed susceptibility to humoral immunity and altered metabolic capacities, while T4P-resistant mutants exhibited increased phagocytosis, suggesting phage resistance may enhance susceptibility to immune responses—a potentially favorable outcome for phage therapy^171^. Advanced clinical trials for CF patients are now being conducted on OligoG CF-5/20, a low molecular mass alginate oligomer that has considerable potential as a new treatment agent. Using advanced imaging techniques and molecular analyses, OligoG was found to inhibit biofilm formation, reduce biomass and EPS, and enhance nanoparticle diffusion and antibiotic efficacy. By disrupting critical DNA-Ca²⁺ interactions within the EPS matrix, OligoG effectively weakens biofilm integrity, positioning it as a promising therapeutic for biofilm-associated infections^172^. OligoG CF-5/20 has proven its capability to modify sputum highly elastic properties, interfere with mucin polymer structures, and dismantle multidrug-resistant *Pseudomonas* biofilms. Prolonged use of inhaled treatments can exert selective evolutionary pressure on bacterial populations residing within lung biofilms. A bead biofilm model was used to expose *P. aeruginosa* to OligoG CF-5/20, either alone or in combination with azithromycin, for 6 weeks in order to examine its long-term effects. The study revealed that prolonged use of OligoG CF-5/20 did not induce substantial genetic adaptations in the bacteria but instead reduced biofilm pathogenicity and improved susceptibility to other antibiotic classes^173^.

### Immunotherapy

KB001-A is a PEGylated monoclonal antibody designed to fight *P. aeruginosa* by disrupting the bacteria’s type III secretion mechanism. It operates by targeting the PcrV protein at the leading edge of the T3SS and effectively inhibiting its action^174,175^. In a 16-week clinical study, KB001-A showed an excellent safety profile, with no notable side effects and was generally well-tolerated. Notably, the therapy reduced the inflammatory marker IL-8 in sputum samples^174^. Another possible strategy is to use anti-Pseudomonas immunoglobulin Y (IgY) antibodies from chicken eggs that have been vaccinated with *P. aeruginosa*. These IgY antibodies target the bacteria’s flagellin, limiting its capacity to attach to epithelial cells and induce pulmonary infections. Individuals with CF gargled with these IgY antibodies in a Phase I trial, and the outcomes were encouraging: no patients experienced long-term colonization, and no negative side effects were observed^176^. The Phase III clinical trial evaluating IgY antibodies against *P. aeruginosa* was discontinued due to unconvincing results. Additionally, panobacumab, a human IgM monoclonal antibody targeting bacterial LPS^177,178^, was evaluated in a Phase IIa trial for nosocomial pneumonia^179^. The trial found panobacumab to be safe, with a lower recurrence rate of pneumonia in the treated patients.

### Vaccine development

Vaccine research provides a viable option to avoid infections and lowering antibiotic abuse, both of which contribute to drug resistance. It was recently proven that a trivalent vaccine consisting of the *P. aeruginosa* exterior membrane proteins (OprF and OprI) and the T3SS translocon protein (PopB), along with or without GM-CSF as an adjuvant, can elicit Th1 and Th2 immune reactions. This vaccine also enhances the secretion of immunoglobulin A (IgA) and induces appropriate levels of various IgG subclasses (G1, G2a, G2b) in burned rat models^180^. To produce a vaccine, it is essential to target the primary virulence elements that enable *P. aeruginosa* to elude the immune system of the host. By promoting cell- and humoral immunity against *P. aeruginosa*, LPS and oligopolysaccharides (OPS) antigens coupled with poly lactic-co-glycolic acid (PLGA) small particles have shown potential as nano vaccines. These conjugates have effectively triggered the production of IgM, IgA, and various IgG subclasses, providing strong immunity in a conjugate-dependent manner^181^. Additionally, as potential vaccine candidates, recombinant proteins that include the entire V-antigen (PcrV) and the C-terminal region of *P. aeruginosa* exoenzyme S (ExoS), as well as adjuvants such as alum and monophosphoryl Lipid A, have been examined. They have effectively boosted humoral and IL-17 responses to safeguard mice from UTI brought on by the *P. aeruginosa* strain PAO1^182^.

A study conducted by ref. ^183^ on the effects of a recombinant vaccine in mice infected with *P. aeruginosa*. The flagellar antigen (reFlgE) used in this vaccine was extracted from the serum of individuals who were recovering from *P. aeruginosa* infections. It has been proven to trigger an immunological response mediated by Th2 cells. The development of anti-reFlgE antibodies in mice greatly decreased the number of bacteria and inflammation.

Furthermore, it has been observed that a whole-cell vaccination that includes nucleic acids and 8-hydroxyguanosine and is inactivated by X-ray irradiation causes a robust humoral immune reaction in DCs. In a mouse model of pneumonia, this strategy has demonstrated effectiveness in avoiding infections caused by both *P. aeruginosa* PAO1 and an MDR clinical isolate W9. Additionally, the vaccination reduces markers of inflammation, including IL-6, IL-8, and TNF-α in DCs while activating the cGAS-STING pathway, modulating TLRs, and inducing cell death, pyroptosis, CD8+ proliferation of T cells, and T1 and T2 cytokine responses^184^.

A purified recombinant fragment of the OprL protein from *P. aeruginosa* has been used as a vaccine to generate a strong pulmonary response, specifically by activating Th17 cells from naïve T cells. The technique provides mice with serotype-independent defence against *P. aeruginosa*-induced acute pulmonary infections^185^. Furthermore, a trivalent DNA vaccine that incorporates *P. aeruginosa* oprL, oprF, and flgE genes has been established, it was found to produce a robust humoral immune response in immunized chickens, as proven by high levels of IFN-γ, IL-2, and IL-4, as well as the proliferation of lymphocytes and protective effectiveness^186^.

Cross-reactive antibodies, which attach to similar antigens in several species, are another way that vaccines might stop infections by other diseases. Mice immunized *with Bordetella pertussis’s* OmpA protein, for example, were shielded from sepsis and pneumonia caused by *P. aeruginosa* PAO1. The study also indicated that the *Bordetella pertussis* whole-cell vaccine decreased *P. aeruginosa* PAO1 colonisation in the respiratory tracts of mice, activated the formation of anti-*P. aeruginosa* IgG and generated antibodies capable of detecting clinical isolates of *P. aeruginosa*^187^. This shows that whole-cell vaccinations targeting a single pathogen may prepare the immune system to respond to other diseases when exposed.

Glycoconjugate vaccines have tremendous potential in tackling many essential human infections because they may induce both T-cell-dependent and T-cell-independent immune reactions^188^. Recombinant proteins with ubiquitous CD4 + T-cell epitopes and the recombinant non-toxic variant of *P. aeruginosa*, exotoxin A are examples of alternate carrier proteins that have been produced to overcome some of these restrictions^189^. Furthermore, the engineering of proteins of the PHA synthesis mechanism in *P. aeruginosa* PAO1 has demonstrated considerable promise in developing vaccine candidates against *P. aeruginosa* infections. Specifically, eliminating critical genes associated with the synthesis of PHA inclusions, alginate, and *Pel* polysaccharide increased the formation of PHA beads, which may then be coated with outer membrane proteins such as AlgE, OprF, and OprI. These PHA beads, when coated with an OprI/F-AlgE fusion antigen, elicited a strong T1-type immunological reaction. Vaccinated mice produced IFN-γ and IgG2c antibodies, and serum antibodies promoted opsonophagocytic death^190^.

Using immunoinformatics technologies, another study forecasted the possible efficacy of an epitope-based vaccination that targets *P. aeruginosa’s* fructose bisphosphate aldolase (FBA) enzyme. This research revealed six promising MHC-I and four MHC-II epitopes for B and T lymphocytes. However, additional in vivo and in vitro studies are required to prove this epitope-based vaccine^191^.

Furthermore, a reverse vaccinology strategy paired with bioinformatics tools was employed to identify 52 possible *P. aeruginosa* antigens that were conserved across genomes from different strains. In mouse models of pneumonia and acute respiratory tract infections, the chosen antigens effectively controlled *P. aeruginosa* infections^192,193^ also developed a novel polyvalent irradiated *P. aeruginosa* vaccine, which contains inactivated pathogens with functional antigenic expression. When administered intranasally, intramuscularly, or subcutaneously, followed by a challenge test, this vaccine demonstrated 95% protective efficacy in a murine model.

### Monoclonal antibodies

Especially for high-risk individuals who are unable to receive vaccinations, monoclonal antibodies (mAbs) are currently used to limit the propagation of *P. aeruginosa* and lessen the severity of infections. These mAbs can provide instant protection, enhancing the benefits of vaccinations. For instance, mAbs have been designed to break up biofilms and prevent antibiotics from successfully targeting *P. aeruginosa* and its co-pathogens, thereby combating the antimicrobial resistance of biofilm-resident bacteria^194^. Antibodies against DNABII protein epitopes and type IV pili from *Haemophilus influenzae*, in particular, dramatically reduced *P. aeruginosa* biofilms and those of other respiratory pathogens such as *Burkholderia cenocepacia*, *Staphylococcus aureus*, *Streptococcus pneumoniae*, and *Moraxella catarrhalis*^194^. Chicken egg yolk immunoglobulins (IgY antibodies) are among the mAbs contenders that have attracted special attention for passive vaccination because of their special qualities. These include having the ability to support stress-free human vaccination techniques, high production rates of antigen-specific antibodies devoid of disease resistance, and a lack of immune-mediated cross-reactivity with mammalian IgG and the complement pathway^157,195^.

Specifically, recombinant PcrV derived from the *P. aeruginosa* PAO1 strain was employed to generate Anti-PcrV IgY antibodies by immunizing hens with the T3SS translocator protein. These Anti-PcrV IgY antibodies enhanced opsonophagocytic killing and inhibited bacterial invasion in rat models of acute pneumonia and burn injuries caused by *P. aeruginosa*^196^. Additionally, recent studies have highlighted the complementary effects of anti-*P. aeruginosa* IgY and β-lactam antibiotics (such as ceftazidime, imipenem, and meropenem), suggesting the potential for combining antibodies with antibiotics to treat complications caused by MDR *P. aeruginosa*^197^.

Monoclonal antibodies, when used alongside antibiotics, can enhance treatment efficacy in cases of severe *P. aeruginosa* pneumonia. In this regard, DNA-delivered monoclonal antibodies (DMAbs)—synthesized in vivo by skeletal muscle tissue and comprising potent human IgG clones along with engineered bispecific IgG1 molecules targeting *P. aeruginosa* strain 6077—have demonstrated protective effects in mice against fatal pneumonia caused by virulent *P. aeruginosa* strains. In addition to preventing pulmonary edema, DMAbs also decreased bacterial colonization in organs like the kidneys and spleen. They also functioned in concert with the widely used carbapenem antibiotic meropenem, were temperature stable, and are thought to be appropriate for treating high-risk patients with chronic illnesses and pathogens that are resistant to a variety of broad-spectrum antibiotics^198^.

Considering their potential, therapeutic monoclonal antibodies have several constraints. For instance, the bivalent human immunoglobulin G1 kappa monoclonal antibody MEDI3902 (gremubamab) could not significantly lower the occurrence of *P. aeruginosa*-associated nosocomial pneumonia among individuals with mechanical ventilation infected with the pathogen^199,200^. Additionally, rabbits were not protected against sepsis by passive vaccination with monoclonal antibodies generated against the chimeric protein pilQ-pilA-DSL region of P. aeruginosa, such as IgY. IgY antibodies’ immunogenicity and protective effectiveness can also vary by dosage and strain. In an acute pneumonia challenge, for example, mice were completely protected against *P. aeruginosa* PAK and PAO1, while animals in a burn wound model were not protected by IgY antibodies produced with engineered type A flagellins of *P. aeruginosa*^201^. In order to prevent bacterial infections, it may be safer to use polyclonal antibody passive vaccination or to directly administer larger dosages of monoclonal antibodies.

## Conclusion

The persistent nature of *P. aeruginosa* biofilms poses a significant challenge to both immune responses and medical interventions. The capacity of biofilms to shield bacteria from immune system cells and antibiotics contributes to persistent infections, particularly in individuals with conditions like CF or chronic wounds. This review emphasizes how biofilms escape innate and adaptive immune responses, resulting in prolonged inflammation and damage to tissues. Although DCs, neutrophils, and macrophages are important components of immune defence, biofilm formations and bacterial virulence factors frequently reduce their efficacy. The establishment of long-term immunity is complicated by the adaptive immune system’s difficulties in removing biofilms, which affect T cells and B cells. In addition, we also observe from our analysis that there is a significant research gap, and Further investigations must be conducted to better characterize the adaptive immune system response in COPD and VAP to fully understand the similarities and differences compared to CF. Innovative therapeutic approaches like immunotherapy, monoclonal antibodies, and vaccines present intriguing possibilities for combating biofilm-associated illnesses, given the drawbacks of existing treatments. In order to help patients with persistent *P. aeruginosa* infections, additional research is required to create specific medicines that strengthen the immune system’s capacity to overcome biofilm resistance.

## Data Availability

No datasets were generated or analyzed during the current study.
